# Crystal structure of [2-({4-[2,6-bis(pyri­din-2-yl)pyri­din-4-yl]phenyl}(methyl)amino)­ethanol-κ^3^ *N*,*N*′,*N*′′]bis­(thio­cyan­ato-κ*N*)zinc *N*,*N*-di­methyl­formamide monosolvate

**DOI:** 10.1107/S1600536814019527

**Published:** 2014-09-06

**Authors:** Shi-Chao Wang, Rou-Chen Pan, Wan-Ying Song, Sheng-Li Li

**Affiliations:** a Department of Chemistry, Anhui University, Hefei 230039, People’s Republic of China, Key Laboratory of Functional Inorganic Materials Chemistry, Hefei 230039, People’s Republic of China

**Keywords:** crystal structure, zinc complex, thio­cyanate ligand, hydrogen bonding, π–π stacking

## Abstract

In the title compound, [Zn(NCS)_2_(C_24_H_22_N_4_O)]·C_3_H_7_NO, the Zn^II^ cation is *N*,*N*′,*N*′′-chelated by one 2-({4-[2,6-bis­(pyridin-2-yl)pyridin-4-yl]phen­yl}(meth­yl)amino)­ethanol ligand and coordinated by two thio­cyanate anions in a distorted N_5_ trigonal–bipyramidal geometry. In the mol­ecule, the three pyridine rings are approximately coplanar [maximum deviation = 0.026 (5) Å], and the mean plane of the three pyridine rings is twisted to the benzene ring with a small dihedral angle of 5.9 (2)°. In the crystal, complex mol­ecules are linked by weak C—H⋯O hydrogen bonds into supra­molecular chains propagated along [110]; π–π stacking is observed between adjacent chains [centroid–centroid distance = 3.678 (4) Å]. The di­methyl­formamide solvent mol­ecules are linked with the complex chains *via* weak C—H⋯O hydrogen bonds.

## Related literature   

For the crystal structures of related Zn^II^ thio­cyanate complexes, see: Nie *et al.* (2014 ▶); Kharat *et al.* (2012 ▶); Eryazici *et al.* (2008 ▶).
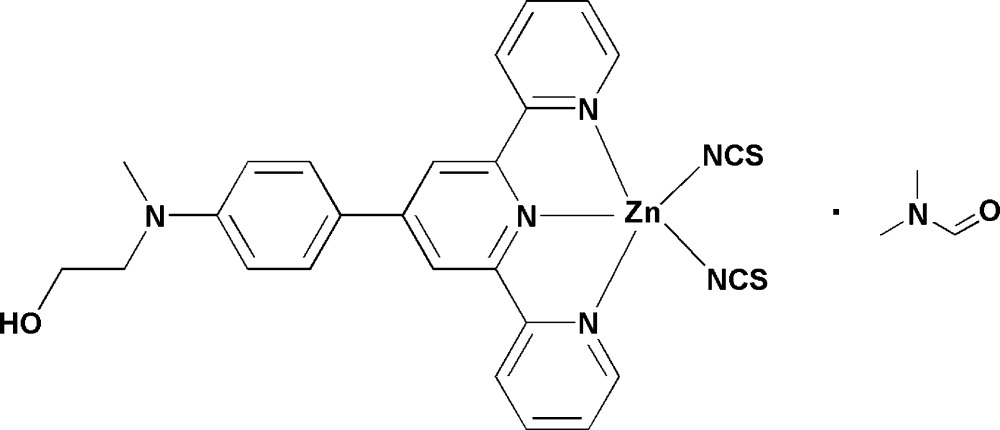


## Experimental   

### Crystal data  

[Zn(NCS)_2_(C_24_H_22_N_4_O)]·C_3_H_7_NO
*M* *_r_* = 637.08
Triclinic, 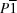

*a* = 9.565 (5) Å
*b* = 12.969 (5) Å
*c* = 13.652 (5) Å
α = 115.656 (5)°
β = 94.646 (5)°
γ = 91.621 (5)°
*V* = 1517.7 (11) Å^3^
*Z* = 2
Mo *K*α radiation
μ = 0.99 mm^−1^
*T* = 298 K
0.30 × 0.20 × 0.20 mm

### Data collection  

Bruker SMART 1000 CCD area-detector diffractometer
Absorption correction: multi-scan (*SADABS*; Bruker, 2002 ▶) *T*_min_ = 0.757, *T*_max_ = 0.827
10881 measured reflections
5293 independent reflections
3729 reflections with *I* > 2σ(*I*)
*R*_int_ = 0.029

### Refinement  

*R*[*F* ^2^ > 2σ(*F* ^2^)] = 0.060
*wR*(*F* ^2^) = 0.174
*S* = 0.99
5293 reflections
373 parameters
H-atom parameters constrained
Δρ_max_ = 0.87 e Å^−3^
Δρ_min_ = −0.72 e Å^−3^

### 

Data collection: *SMART* (Bruker, 2007 ▶); cell refinement: *SAINT* (Bruker, 2007 ▶); data reduction: *SAINT*; program(s) used to solve structure: *SHELXTL* (Sheldrick, 2008 ▶); program(s) used to refine structure: *SHELXTL*; molecular graphics: *SHELXTL*; software used to prepare material for publication: *SHELXTL*.

## Supplementary Material

Crystal structure: contains datablock(s) I, Global. DOI: 10.1107/S1600536814019527/xu5817sup1.cif

Structure factors: contains datablock(s) I. DOI: 10.1107/S1600536814019527/xu5817Isup2.hkl

**Fig. 1.**
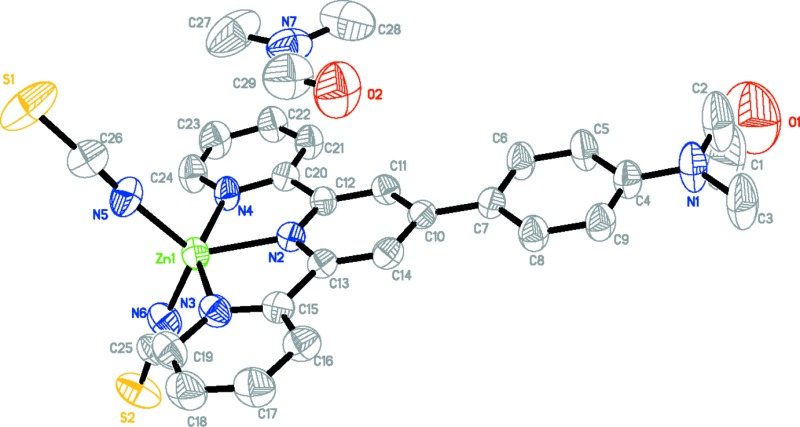
The molecular structure of the title compound, with the atom-numbering scheme. Displacement ellipsoids are drawn at the 50% probability level. H atoms have been omitted.

. DOI: 10.1107/S1600536814019527/xu5817fig1.tif
The mol­ecular structure of the title compound, with the atom-numbering scheme. Displacement ellipsoids are drawn at the 50% probability level. H atoms have been omitted.

**Fig. 2.**
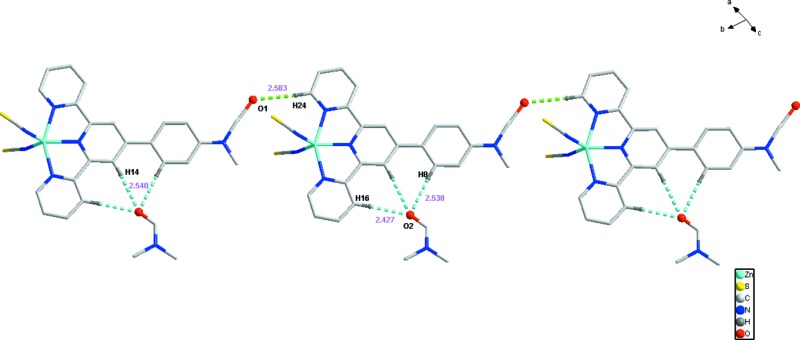
The one-dimensional chain structure of the title compound, Hydrogen atoms are omitted for clarity.

. DOI: 10.1107/S1600536814019527/xu5817fig2.tif
The one-dimensional chain structure of the title compound, Hydrogen atoms are omitted for clarity.

**Fig. 3.**
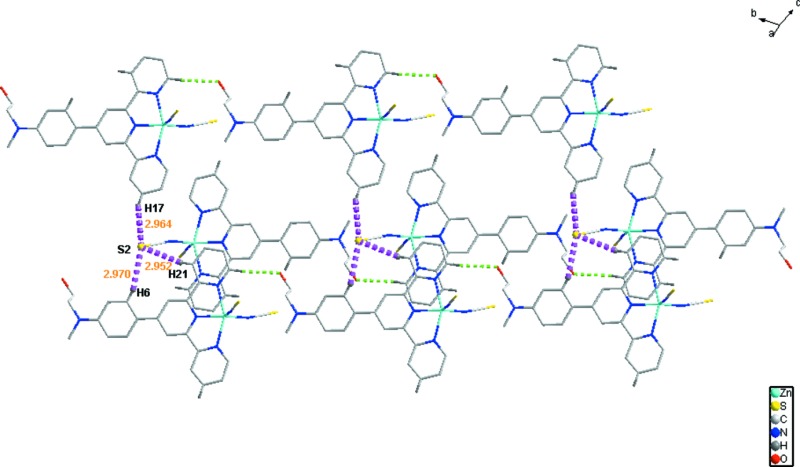
The two-dimensional networks of the title compound, Hydrogen atoms are omitted for clarity.

. DOI: 10.1107/S1600536814019527/xu5817fig3.tif
The two-dimensional networks of the title compound, Hydrogen atoms are omitted for clarity.

CCDC reference: 1020385

Additional supporting information:  crystallographic information; 3D view; checkCIF report

## Figures and Tables

**Table 1:** Selected bond lengths (Å)

Zn1—N2	2.051 (4)
Zn1—N3	2.163 (4)
Zn1—N4	2.224 (4)
Zn1—N5	1.953 (5)
Zn1—N6	1.969 (4)

**Table 2:** Hydrogen-bond geometry (Å, °)

*D*—H⋯*A*	*D*—H	H⋯*A*	*D*⋯*A*	*D*—H⋯*A*
C8—H8⋯O2^i^	0.93	2.54	3.457 (8)	170
C14—H14⋯O2^i^	0.93	2.54	3.470 (7)	177
C16—H16⋯O2^i^	0.93	2.43	3.356 (9)	178
C24—H24⋯O1^ii^	0.93	2.58	3.488 (13)	164

Symmetry codes: (i) 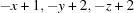; (ii) 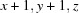.

## supplementary crystallographic information

### S1. Comment

Zinc complexes with terpyridine derivatives are currently attracting attention for their interesting molecular topologies and crystal packing motifs, as well as the fact that they may be designed with specific applications in bioinorganic chemistry and material (Nie *et al.*, 2014; Kharat *et al.*, 2012). In turn, countless Zn terpyridine complexes have been surveyed and their crystals were demonstrated. However, the crystal of Zn(SCN)_2_ terpyridine complexes are rarely mentioned (Eryazici *et al.*, 2008). In this paper, we report the crystal structure of the terpyridine- Zinc complex. The molecular structure with the numbering scheme is shown in Fig. 1, the Zn^II^ is coordinated by three N atoms from terpyridine ligand and two thiocyanate anions in a highly distorted square-pyramidal trigonal bipyramidal geometry. Bond distances and angles around the Zn^II^ center are in the range 1.954 (5)–2.224 (4) Å and 74.93 (14)–124.48 (17)°, respectively. The dihedral angle between pyridine ring and phenyl ring is 6.24°, which can ascribe to the existence of the dimethylformamide molecule that restricts the rotation of single bonds and increase molecule planarity by C— H···O interactions. The one-dimensional chains are assembled by C24—H24···O1 (2.583 Å) hydrogen bond between adjacent complex molecules (Fig.2). In addition, two-dimensional networks formed by multiply C—H···S hydrogen bonds. The distances of C6—H6··· S2, C21—H21···S2 and C17—H17···S2 are nearly the same, which are 2.970 Å, 2.952 Å and 2.964 Å, respectively (Fig.3).

### S2. Experimental

To a solution of Zn(SCN)_2_ (0.18 g, 1 mmol) in freshly distilled ethanol (50 ml), was added *N*-methyl-*N*-(4-(2,6-di(pyridin-2-yl)pyridin-4-yl)phenyl)amino)ethanol (0.38 g, 1 mmol). The mixture was refluxed for 4 h. Then resulted orange suspension was filtered, washed with ethanol and dried *in vacuo*. Subsequent diffusion of diethyl ether into the concentrated dimethylformamide solution gave complex as air-stable orange crystals.

### S3. Refinement

All hydrogen atoms were placed in geometrically idealized positions and constrained to ride on their parent atoms, with O—H = 0.96 and C—H = 0.93–0.97 Å, *U*_iso_(H) = 1.5*U*_eq_(O,C) for hydroxyl and methyl H atoms and 1.2*U*_eq_(C) for the others.

### Crystal data

**Table:**

[Zn(NCS)_2_(C_24_H_22_N_4_O)]·C_3_H_7_NO	*Z* = 2
*M**_r_* = 637.08	*F*(000) = 660
Triclinic, *P*1	*D*_x_ = 1.394 Mg m^−^^3^
Hall symbol: -P 1	Mo *K*α radiation, λ = 0.71069 Å
*a* = 9.565 (5) Å	Cell parameters from 2689 reflections
*b* = 12.969 (5) Å	θ = 2.6–21.8°
*c* = 13.652 (5) Å	µ = 0.99 mm^−^^1^
α = 115.656 (5)°	*T* = 298 K
β = 94.646 (5)°	Block, orange
γ = 91.621 (5)°	0.30 × 0.20 × 0.20 mm
*V* = 1517.7 (11) Å^3^	

### Data collection

**Table:**

Bruker SMART 1000 CCD area-detector diffractometer	5293 independent reflections
Radiation source: fine-focus sealed tube	3729 reflections with *I* > 2σ(*I*)
Graphite monochromator	*R*_int_ = 0.029
phi and ω scans	θ_max_ = 25.0°, θ_min_ = 1.7°
Absorption correction: multi-scan (*SADABS*; Bruker, 2002)	*h* = −11→11
*T*_min_ = 0.757, *T*_max_ = 0.827	*k* = −15→15
10881 measured reflections	*l* = −14→16

### Refinement

**Table:**

Refinement on *F*^2^	Primary atom site location: structure-invariant direct methods
Least-squares matrix: full	Secondary atom site location: difference Fourier map
*R*[*F*^2^ > 2σ(*F*^2^)] = 0.060	Hydrogen site location: inferred from neighbouring sites
*wR*(*F*^2^) = 0.174	H-atom parameters constrained
*S* = 0.99	*w* = 1/[σ^2^(*F*_o_^2^) + (0.0928*P*)^2^ + 1.3834*P*] where *P* = (*F*_o_^2^ + 2*F*_c_^2^)/3
5293 reflections	(Δ/σ)_max_ = 0.006
373 parameters	Δρ_max_ = 0.87 e Å^−^^3^
0 restraints	Δρ_min_ = −0.72 e Å^−^^3^

### Special details

**Table:**

Geometry. All e.s.d.'s (except the e.s.d. in the dihedral angle between two l.s. planes) are estimated using the full covariance matrix. The cell e.s.d.'s are taken into account individually in the estimation of e.s.d.'s in distances, angles and torsion angles; correlations between e.s.d.'s in cell parameters are only used when they are defined by crystal symmetry. An approximate (isotropic) treatment of cell e.s.d.'s is used for estimating e.s.d.'s involving l.s. planes.
Refinement. Refinement of *F*^2^ against ALL reflections. The weighted *R*-factor *wR* and goodness of fit *S* are based on *F*^2^, conventional *R*-factors *R* are based on *F*, with *F* set to zero for negative *F*^2^. The threshold expression of *F*^2^ > σ(*F*^2^) is used only for calculating *R*-factors(gt) *etc*. and is not relevant to the choice of reflections for refinement. *R*-factors based on *F*^2^ are statistically about twice as large as those based on *F*, and *R*- factors based on ALL data will be even larger.

### Fractional atomic coordinates and isotropic or equivalent isotropic displacement parameters (Å^2^)

**Table:**

	*x*	*y*	*z*	*U*_iso_*/*U*_eq_	
Zn1	0.69718 (6)	1.21976 (4)	0.76963 (4)	0.0543 (2)	
S2	0.5329 (2)	1.42626 (13)	0.57934 (12)	0.0919 (6)	
S1	1.18423 (19)	1.2588 (2)	0.87203 (16)	0.1314 (9)	
C11	0.4729 (5)	0.8990 (4)	0.6857 (3)	0.0493 (10)	
H11	0.4628	0.8285	0.6244	0.059*	
C7	0.3121 (5)	0.8217 (4)	0.7783 (4)	0.0508 (11)	
N4	0.7085 (4)	1.0716 (3)	0.6082 (3)	0.0570 (10)	
C10	0.4020 (4)	0.9141 (4)	0.7772 (3)	0.0473 (10)	
N2	0.5729 (4)	1.0896 (3)	0.7721 (3)	0.0462 (8)	
C21	0.6321 (5)	0.8772 (4)	0.4976 (4)	0.0616 (13)	
H21	0.5795	0.8117	0.4886	0.074*	
C20	0.6351 (5)	0.9760 (4)	0.5926 (3)	0.0474 (10)	
C15	0.5317 (5)	1.2277 (4)	0.9491 (4)	0.0509 (11)	
C14	0.4221 (5)	1.0233 (4)	0.8652 (4)	0.0514 (11)	
H14	0.3781	1.0387	0.9280	0.062*	
C25	0.5934 (6)	1.3671 (4)	0.6558 (4)	0.0601 (12)	
N3	0.6160 (4)	1.2966 (3)	0.9271 (3)	0.0552 (9)	
C13	0.5056 (4)	1.1074 (4)	0.8597 (3)	0.0464 (10)	
C12	0.5556 (4)	0.9861 (4)	0.6862 (3)	0.0458 (10)	
C5	0.2196 (6)	0.6233 (4)	0.6932 (4)	0.0746 (16)	
H5	0.2168	0.5511	0.6346	0.089*	
C4	0.1361 (5)	0.6400 (4)	0.7783 (4)	0.0641 (13)	
C8	0.2290 (5)	0.8372 (4)	0.8625 (4)	0.0595 (12)	
H8	0.2308	0.9098	0.9205	0.071*	
N1	0.0541 (6)	0.5523 (4)	0.7781 (4)	0.0904 (16)	
C9	0.1450 (5)	0.7506 (4)	0.8635 (4)	0.0636 (13)	
H9	0.0928	0.7657	0.9222	0.076*	
C16	0.4713 (5)	1.2637 (4)	1.0458 (4)	0.0632 (13)	
H16	0.4126	1.2139	1.0592	0.076*	
C22	0.7081 (6)	0.8762 (5)	0.4153 (4)	0.0701 (14)	
H22	0.7072	0.8101	0.3500	0.084*	
C6	0.3040 (6)	0.7096 (4)	0.6943 (4)	0.0671 (14)	
H6	0.3589	0.6938	0.6370	0.081*	
C17	0.5010 (6)	1.3769 (5)	1.1227 (4)	0.0736 (15)	
H17	0.4627	1.4041	1.1891	0.088*	
C26	1.0166 (7)	1.2465 (5)	0.8394 (4)	0.0734 (15)	
C23	0.7846 (6)	0.9739 (5)	0.4315 (5)	0.0787 (16)	
H23	0.8370	0.9751	0.3775	0.094*	
C18	0.5873 (6)	1.4481 (5)	1.0997 (5)	0.0775 (16)	
H18	0.6069	1.5244	1.1499	0.093*	
C24	0.7831 (6)	1.0690 (5)	0.5274 (5)	0.0733 (15)	
H24	0.8356	1.1351	0.5379	0.088*	
N6	0.6325 (5)	1.3253 (4)	0.7093 (4)	0.0690 (11)	
C19	0.6443 (6)	1.4058 (4)	1.0021 (4)	0.0687 (14)	
H19	0.7042	1.4538	0.9874	0.082*	
O2	0.7459 (6)	0.9090 (5)	0.9008 (4)	0.1267 (19)	
O1	−0.0642 (12)	0.3133 (8)	0.5213 (8)	0.253 (5)	
H1C	−0.1604	0.2942	0.4900	0.379*	
C3	−0.0373 (8)	0.5720 (6)	0.8653 (6)	0.111 (3)	
H3A	0.0188	0.6023	0.9346	0.166*	
H3B	−0.0860	0.5009	0.8525	0.166*	
H3C	−0.1045	0.6258	0.8657	0.166*	
N7	0.8572 (6)	0.8651 (5)	0.7513 (5)	0.0909 (16)	
N5	0.8987 (5)	1.2422 (4)	0.8163 (4)	0.0756 (13)	
C29	0.8300 (9)	0.9318 (8)	0.8483 (7)	0.114 (2)	
H29	0.8783	1.0037	0.8819	0.137*	
C2	0.0517 (11)	0.4346 (7)	0.6910 (7)	0.129 (3)	
H2A	0.1441	0.4184	0.6657	0.155*	
H2B	0.0240	0.3797	0.7177	0.155*	
C28	0.7953 (10)	0.7508 (8)	0.6975 (8)	0.141 (3)	
H28A	0.8676	0.6985	0.6717	0.212*	
H28B	0.7307	0.7431	0.6368	0.212*	
H28C	0.7459	0.7337	0.7478	0.212*	
C1	−0.0503 (12)	0.4279 (10)	0.6026 (10)	0.182 (5)	
H1A	−0.1403	0.4510	0.6300	0.219*	
H1B	−0.0184	0.4786	0.5724	0.219*	
C27	0.9575 (8)	0.8978 (9)	0.6971 (8)	0.144 (4)	
H27A	0.9899	0.9767	0.7405	0.216*	
H27B	0.9148	0.8882	0.6273	0.216*	
H27C	1.0356	0.8506	0.6868	0.216*	

### Atomic displacement parameters (Å^2^)

**Table:**

	*U*^11^	*U*^22^	*U*^33^	*U*^12^	*U*^13^	*U*^23^
Zn1	0.0543 (4)	0.0515 (3)	0.0586 (4)	−0.0060 (2)	−0.0007 (2)	0.0270 (3)
S2	0.1544 (17)	0.0717 (9)	0.0591 (9)	0.0319 (10)	0.0163 (9)	0.0352 (8)
S1	0.0642 (11)	0.216 (3)	0.0843 (12)	0.0322 (13)	0.0012 (9)	0.0380 (14)
C11	0.058 (3)	0.044 (2)	0.040 (2)	0.001 (2)	0.006 (2)	0.0134 (19)
C7	0.048 (3)	0.052 (3)	0.052 (3)	−0.001 (2)	0.007 (2)	0.022 (2)
N4	0.059 (2)	0.058 (2)	0.056 (2)	−0.0086 (19)	0.0118 (19)	0.0269 (19)
C10	0.046 (2)	0.049 (2)	0.048 (3)	0.0038 (19)	0.0056 (19)	0.022 (2)
N2	0.048 (2)	0.048 (2)	0.0406 (19)	0.0012 (16)	0.0037 (16)	0.0170 (17)
C21	0.068 (3)	0.065 (3)	0.049 (3)	−0.006 (2)	0.013 (2)	0.021 (2)
C20	0.049 (2)	0.052 (3)	0.041 (2)	0.002 (2)	0.0045 (19)	0.020 (2)
C15	0.052 (3)	0.051 (3)	0.044 (3)	0.003 (2)	−0.002 (2)	0.018 (2)
C14	0.052 (3)	0.059 (3)	0.042 (2)	0.006 (2)	0.012 (2)	0.020 (2)
C25	0.078 (3)	0.049 (3)	0.048 (3)	0.006 (2)	0.013 (2)	0.015 (2)
N3	0.062 (2)	0.049 (2)	0.050 (2)	0.0015 (18)	−0.0014 (18)	0.0191 (18)
C13	0.045 (2)	0.049 (2)	0.043 (2)	0.0040 (19)	0.0044 (19)	0.018 (2)
C12	0.048 (2)	0.047 (2)	0.045 (2)	0.0013 (19)	0.0058 (19)	0.023 (2)
C5	0.096 (4)	0.055 (3)	0.066 (3)	−0.014 (3)	0.030 (3)	0.017 (3)
C4	0.071 (3)	0.064 (3)	0.060 (3)	−0.009 (3)	0.014 (3)	0.029 (3)
C8	0.068 (3)	0.053 (3)	0.052 (3)	−0.005 (2)	0.015 (2)	0.018 (2)
N1	0.115 (4)	0.072 (3)	0.072 (3)	−0.035 (3)	0.029 (3)	0.019 (3)
C9	0.071 (3)	0.067 (3)	0.055 (3)	−0.002 (3)	0.024 (2)	0.026 (3)
C16	0.066 (3)	0.065 (3)	0.051 (3)	0.007 (2)	0.008 (2)	0.016 (2)
C22	0.084 (4)	0.069 (3)	0.053 (3)	−0.005 (3)	0.019 (3)	0.021 (3)
C6	0.077 (4)	0.062 (3)	0.060 (3)	−0.008 (3)	0.026 (3)	0.022 (3)
C17	0.088 (4)	0.065 (3)	0.051 (3)	0.006 (3)	0.009 (3)	0.009 (3)
C26	0.074 (4)	0.091 (4)	0.049 (3)	0.013 (3)	0.009 (3)	0.024 (3)
C23	0.090 (4)	0.090 (4)	0.065 (4)	−0.001 (3)	0.029 (3)	0.038 (3)
C18	0.088 (4)	0.057 (3)	0.062 (3)	0.002 (3)	−0.003 (3)	0.004 (3)
C24	0.079 (4)	0.075 (4)	0.070 (4)	−0.013 (3)	0.014 (3)	0.035 (3)
N6	0.086 (3)	0.059 (3)	0.066 (3)	0.002 (2)	0.001 (2)	0.032 (2)
C19	0.081 (4)	0.050 (3)	0.065 (3)	−0.003 (3)	−0.001 (3)	0.017 (3)
O2	0.142 (5)	0.142 (5)	0.098 (4)	−0.003 (4)	0.047 (3)	0.050 (3)
O1	0.304 (13)	0.181 (8)	0.201 (9)	−0.063 (8)	0.005 (8)	0.024 (7)
C3	0.141 (6)	0.099 (5)	0.088 (5)	−0.033 (4)	0.046 (4)	0.035 (4)
N7	0.090 (4)	0.110 (4)	0.090 (4)	0.039 (3)	0.033 (3)	0.054 (4)
N5	0.054 (3)	0.090 (3)	0.081 (3)	−0.010 (2)	−0.005 (2)	0.038 (3)
C29	0.125 (7)	0.135 (7)	0.095 (6)	0.028 (5)	0.032 (5)	0.058 (5)
C2	0.177 (9)	0.112 (6)	0.097 (6)	−0.048 (6)	0.042 (6)	0.042 (5)
C28	0.159 (9)	0.111 (7)	0.146 (8)	0.044 (6)	0.004 (6)	0.051 (6)
C1	0.167 (10)	0.183 (11)	0.143 (9)	−0.081 (9)	0.005 (8)	0.029 (9)
C27	0.090 (5)	0.252 (12)	0.157 (8)	0.045 (6)	0.039 (5)	0.145 (8)

### Geometric parameters (Å, º)

**Table:**

Zn1—N2	2.051 (4)	N1—C3	1.472 (7)
Zn1—N3	2.163 (4)	C9—H9	0.9300
Zn1—N4	2.224 (4)	C16—C17	1.389 (7)
Zn1—N5	1.953 (5)	C16—H16	0.9300
Zn1—N6	1.969 (4)	C22—C23	1.367 (8)
S2—C25	1.623 (6)	C22—H22	0.9300
S1—C26	1.613 (7)	C6—H6	0.9300
C11—C12	1.359 (6)	C17—C18	1.372 (8)
C11—C10	1.410 (6)	C17—H17	0.9300
C11—H11	0.9300	C26—N5	1.139 (7)
C7—C8	1.395 (6)	C23—C24	1.357 (8)
C7—C6	1.404 (7)	C23—H23	0.9300
C7—C10	1.462 (6)	C18—C19	1.369 (8)
N4—C20	1.331 (6)	C18—H18	0.9300
N4—C24	1.351 (6)	C24—H24	0.9300
C10—C14	1.401 (6)	C19—H19	0.9300
N2—C13	1.339 (5)	O2—C29	1.229 (8)
N2—C12	1.341 (5)	O1—C1	1.412 (12)
C21—C20	1.370 (6)	O1—H1C	0.9600
C21—C22	1.382 (7)	C3—H3A	0.9600
C21—H21	0.9300	C3—H3B	0.9600
C20—C12	1.499 (6)	C3—H3C	0.9600
C15—N3	1.330 (6)	N7—C29	1.283 (9)
C15—C16	1.378 (6)	N7—C27	1.418 (8)
C15—C13	1.505 (6)	N7—C28	1.423 (10)
C14—C13	1.366 (6)	C29—H29	0.9300
C14—H14	0.9300	C2—C1	1.459 (13)
C25—N6	1.130 (6)	C2—H2A	0.9700
N3—C19	1.343 (6)	C2—H2B	0.9700
C5—C6	1.355 (7)	C28—H28A	0.9600
C5—C4	1.405 (7)	C28—H28B	0.9600
C5—H5	0.9300	C28—H28C	0.9600
C4—N1	1.362 (6)	C1—H1A	0.9700
C4—C9	1.397 (7)	C1—H1B	0.9700
C8—C9	1.368 (6)	C27—H27A	0.9600
C8—H8	0.9300	C27—H27B	0.9600
N1—C2	1.471 (9)	C27—H27C	0.9600
			
N5—Zn1—N6	113.0 (2)	C15—C16—H16	121.1
N5—Zn1—N2	122.32 (17)	C17—C16—H16	121.1
N6—Zn1—N2	124.49 (17)	C23—C22—C21	118.9 (5)
N5—Zn1—N3	99.89 (17)	C23—C22—H22	120.6
N6—Zn1—N3	100.04 (16)	C21—C22—H22	120.6
N2—Zn1—N3	75.96 (14)	C5—C6—C7	122.8 (5)
N5—Zn1—N4	96.67 (17)	C5—C6—H6	118.6
N6—Zn1—N4	95.28 (16)	C7—C6—H6	118.6
N2—Zn1—N4	74.93 (14)	C18—C17—C16	119.4 (5)
N3—Zn1—N4	150.86 (15)	C18—C17—H17	120.3
C12—C11—C10	120.8 (4)	C16—C17—H17	120.3
C12—C11—H11	119.6	N5—C26—S1	177.1 (6)
C10—C11—H11	119.6	C24—C23—C22	119.2 (5)
C8—C7—C6	114.9 (4)	C24—C23—H23	120.4
C8—C7—C10	123.1 (4)	C22—C23—H23	120.4
C6—C7—C10	122.0 (4)	C19—C18—C17	119.3 (5)
C20—N4—C24	118.1 (4)	C19—C18—H18	120.3
C20—N4—Zn1	115.1 (3)	C17—C18—H18	120.3
C24—N4—Zn1	126.8 (3)	N4—C24—C23	122.6 (5)
C14—C10—C11	115.6 (4)	N4—C24—H24	118.7
C14—C10—C7	122.6 (4)	C23—C24—H24	118.7
C11—C10—C7	121.7 (4)	C25—N6—Zn1	166.5 (4)
C13—N2—C12	118.7 (4)	N3—C19—C18	121.7 (5)
C13—N2—Zn1	120.2 (3)	N3—C19—H19	119.1
C12—N2—Zn1	121.1 (3)	C18—C19—H19	119.1
C20—C21—C22	119.1 (5)	C1—O1—H1C	109.1
C20—C21—H21	120.4	N1—C3—H3A	109.5
C22—C21—H21	120.4	N1—C3—H3B	109.5
N4—C20—C21	122.1 (4)	H3A—C3—H3B	109.5
N4—C20—C12	114.6 (4)	N1—C3—H3C	109.5
C21—C20—C12	123.2 (4)	H3A—C3—H3C	109.5
N3—C15—C16	122.7 (4)	H3B—C3—H3C	109.5
N3—C15—C13	115.0 (4)	C29—N7—C27	122.1 (8)
C16—C15—C13	122.3 (4)	C29—N7—C28	120.4 (7)
C13—C14—C10	120.5 (4)	C27—N7—C28	117.3 (8)
C13—C14—H14	119.7	C26—N5—Zn1	174.8 (5)
C10—C14—H14	119.7	O2—C29—N7	125.8 (9)
N6—C25—S2	178.5 (5)	O2—C29—H29	117.1
C15—N3—C19	118.9 (4)	N7—C29—H29	117.1
C15—N3—Zn1	115.7 (3)	C1—C2—N1	106.5 (9)
C19—N3—Zn1	125.4 (4)	C1—C2—H2A	110.4
N2—C13—C14	122.3 (4)	N1—C2—H2A	110.4
N2—C13—C15	113.2 (4)	C1—C2—H2B	110.4
C14—C13—C15	124.5 (4)	N1—C2—H2B	110.4
N2—C12—C11	122.0 (4)	H2A—C2—H2B	108.6
N2—C12—C20	114.2 (4)	N7—C28—H28A	109.5
C11—C12—C20	123.7 (4)	N7—C28—H28B	109.5
C6—C5—C4	121.8 (5)	H28A—C28—H28B	109.5
C6—C5—H5	119.1	N7—C28—H28C	109.5
C4—C5—H5	119.1	H28A—C28—H28C	109.5
N1—C4—C9	122.4 (5)	H28B—C28—H28C	109.5
N1—C4—C5	121.6 (5)	O1—C1—C2	108.1 (11)
C9—C4—C5	116.1 (4)	O1—C1—H1A	110.1
C9—C8—C7	123.0 (4)	C2—C1—H1A	110.1
C9—C8—H8	118.5	O1—C1—H1B	110.1
C7—C8—H8	118.5	C2—C1—H1B	110.1
C4—N1—C2	121.9 (5)	H1A—C1—H1B	108.4
C4—N1—C3	121.0 (5)	N7—C27—H27A	109.5
C2—N1—C3	117.1 (5)	N7—C27—H27B	109.5
C8—C9—C4	121.4 (4)	H27A—C27—H27B	109.5
C8—C9—H9	119.3	N7—C27—H27C	109.5
C4—C9—H9	119.3	H27A—C27—H27C	109.5
C15—C16—C17	117.9 (5)	H27B—C27—H27C	109.5
			
N5—Zn1—N4—C20	121.1 (3)	C13—N2—C12—C11	−0.5 (6)
N6—Zn1—N4—C20	−124.9 (3)	Zn1—N2—C12—C11	−179.5 (3)
N2—Zn1—N4—C20	−0.6 (3)	C13—N2—C12—C20	179.7 (4)
N3—Zn1—N4—C20	−3.3 (5)	Zn1—N2—C12—C20	0.7 (5)
N5—Zn1—N4—C24	−57.5 (5)	C10—C11—C12—N2	−0.5 (7)
N6—Zn1—N4—C24	56.4 (5)	C10—C11—C12—C20	179.4 (4)
N2—Zn1—N4—C24	−179.3 (5)	N4—C20—C12—N2	−1.2 (6)
N3—Zn1—N4—C24	178.1 (4)	C21—C20—C12—N2	179.6 (4)
C12—C11—C10—C14	0.7 (6)	N4—C20—C12—C11	179.0 (4)
C12—C11—C10—C7	179.8 (4)	C21—C20—C12—C11	−0.2 (7)
C8—C7—C10—C14	6.0 (7)	C6—C5—C4—N1	−178.4 (6)
C6—C7—C10—C14	−173.5 (5)	C6—C5—C4—C9	0.2 (9)
C8—C7—C10—C11	−173.0 (4)	C6—C7—C8—C9	−0.4 (8)
C6—C7—C10—C11	7.5 (7)	C10—C7—C8—C9	−179.9 (5)
N5—Zn1—N2—C13	92.5 (4)	C9—C4—N1—C2	−176.4 (7)
N6—Zn1—N2—C13	−93.1 (3)	C5—C4—N1—C2	2.1 (10)
N3—Zn1—N2—C13	−0.4 (3)	C9—C4—N1—C3	4.1 (9)
N4—Zn1—N2—C13	−179.1 (3)	C5—C4—N1—C3	−177.4 (6)
N5—Zn1—N2—C12	−88.5 (4)	C7—C8—C9—C4	−0.8 (8)
N6—Zn1—N2—C12	85.9 (4)	N1—C4—C9—C8	179.5 (6)
N3—Zn1—N2—C12	178.6 (3)	C5—C4—C9—C8	0.9 (8)
N4—Zn1—N2—C12	−0.1 (3)	N3—C15—C16—C17	0.0 (7)
C24—N4—C20—C21	−0.9 (7)	C13—C15—C16—C17	−179.4 (4)
Zn1—N4—C20—C21	−179.7 (4)	C20—C21—C22—C23	0.3 (8)
C24—N4—C20—C12	179.9 (4)	C4—C5—C6—C7	−1.5 (10)
Zn1—N4—C20—C12	1.1 (5)	C8—C7—C6—C5	1.5 (8)
C22—C21—C20—N4	0.4 (7)	C10—C7—C6—C5	−179.0 (5)
C22—C21—C20—C12	179.5 (4)	C15—C16—C17—C18	0.4 (8)
C11—C10—C14—C13	0.0 (6)	C21—C22—C23—C24	−0.4 (9)
C7—C10—C14—C13	−179.1 (4)	C16—C17—C18—C19	−1.0 (9)
C16—C15—N3—C19	0.2 (7)	C20—N4—C24—C23	0.8 (8)
C13—C15—N3—C19	179.6 (4)	Zn1—N4—C24—C23	179.4 (4)
C16—C15—N3—Zn1	−179.9 (4)	C22—C23—C24—N4	−0.2 (9)
C13—C15—N3—Zn1	−0.5 (5)	S2—C25—N6—Zn1	87 (18)
N5—Zn1—N3—C15	−120.5 (3)	N5—Zn1—N6—C25	107 (2)
N6—Zn1—N3—C15	123.8 (3)	N2—Zn1—N6—C25	−67 (2)
N2—Zn1—N3—C15	0.5 (3)	N3—Zn1—N6—C25	−147 (2)
N4—Zn1—N3—C15	3.2 (5)	N4—Zn1—N6—C25	8 (2)
N5—Zn1—N3—C19	59.3 (4)	C15—N3—C19—C18	−0.8 (8)
N6—Zn1—N3—C19	−56.3 (4)	Zn1—N3—C19—C18	179.3 (4)
N2—Zn1—N3—C19	−179.6 (4)	C17—C18—C19—N3	1.2 (9)
N4—Zn1—N3—C19	−177.0 (4)	S1—C26—N5—Zn1	148 (8)
C12—N2—C13—C14	1.2 (6)	N6—Zn1—N5—C26	−133 (5)
Zn1—N2—C13—C14	−179.8 (3)	N2—Zn1—N5—C26	42 (5)
C12—N2—C13—C15	−178.7 (4)	N3—Zn1—N5—C26	122 (5)
Zn1—N2—C13—C15	0.3 (5)	N4—Zn1—N5—C26	−34 (5)
C10—C14—C13—N2	−1.0 (7)	C27—N7—C29—O2	−179.2 (7)
C10—C14—C13—C15	179.0 (4)	C28—N7—C29—O2	−4.5 (12)
N3—C15—C13—N2	0.2 (5)	C4—N1—C2—C1	−85.3 (9)
C16—C15—C13—N2	179.6 (4)	C3—N1—C2—C1	94.2 (8)
N3—C15—C13—C14	−179.7 (4)	N1—C2—C1—O1	−175.1 (7)
C16—C15—C13—C14	−0.3 (7)		

### Hydrogen-bond geometry (Å, º)

**Table:**

*D*—H···*A*	*D*—H	H···*A*	*D*···*A*	*D*—H···*A*
C8—H8···O2^i^	0.93	2.54	3.457 (8)	170
C14—H14···O2^i^	0.93	2.54	3.470 (7)	177
C16—H16···O2^i^	0.93	2.43	3.356 (9)	178
C24—H24···O1^ii^	0.93	2.58	3.488 (13)	164

Symmetry codes: (i) −*x*+1, −*y*+2, −*z*+2; (ii) *x*+1, *y*+1, *z*.
