# Supplementary figures and images for: Crystal structure of [2-({4-[2,6-bis(pyri­din-2-yl)pyri­din-4-yl]phenyl}(methyl)amino)­ethanol-κ3 N,N′,N′′]bis­(thio­cyan­ato-κN)zinc N,N-di­methyl­formamide monosolvate
Source: Acta Crystallogr Sect E Struct Rep Online. 2014 Sep 6;70(Pt 10):m347–8. doi: 10.1107/S1600536814019527 (PMC4257219; doi:10.1107/S1600536814019527)

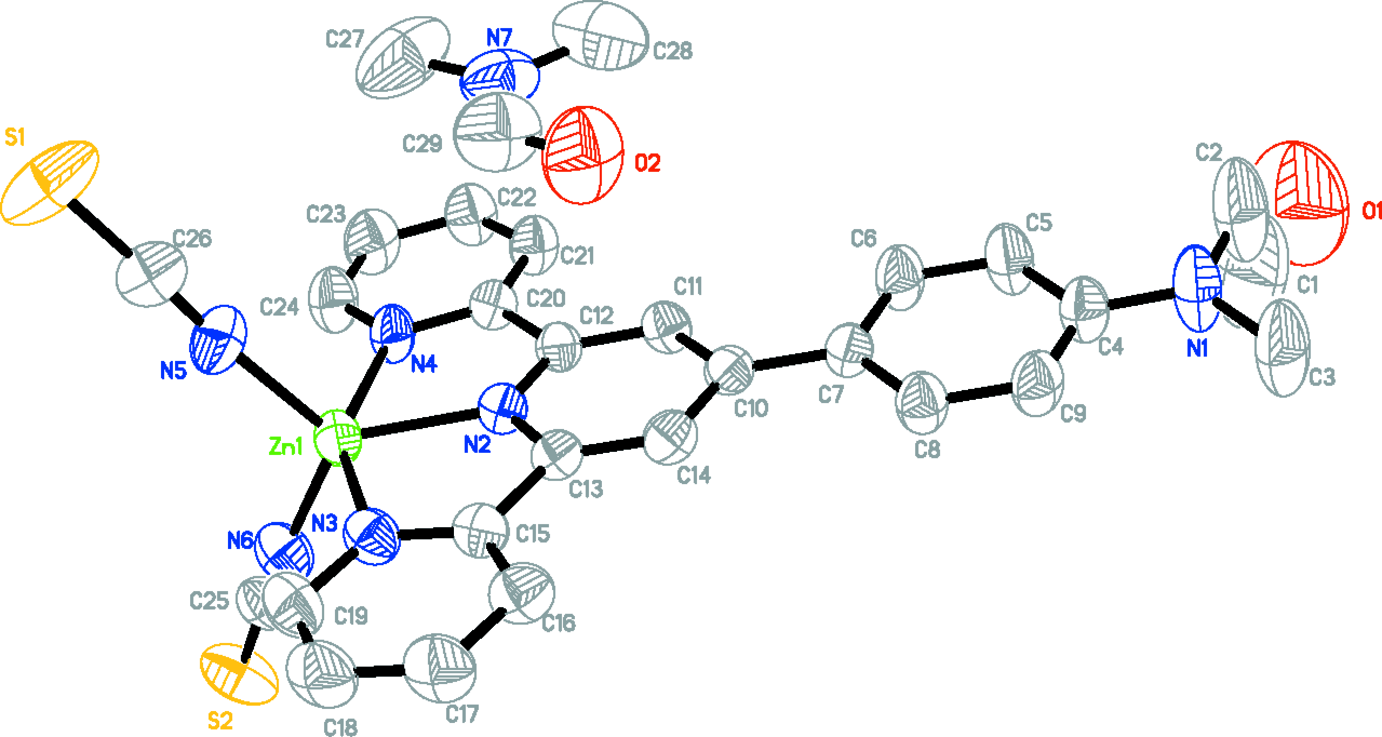

Supplement: Supplementary file 3 [file e-70-0m347-fig1.tif]

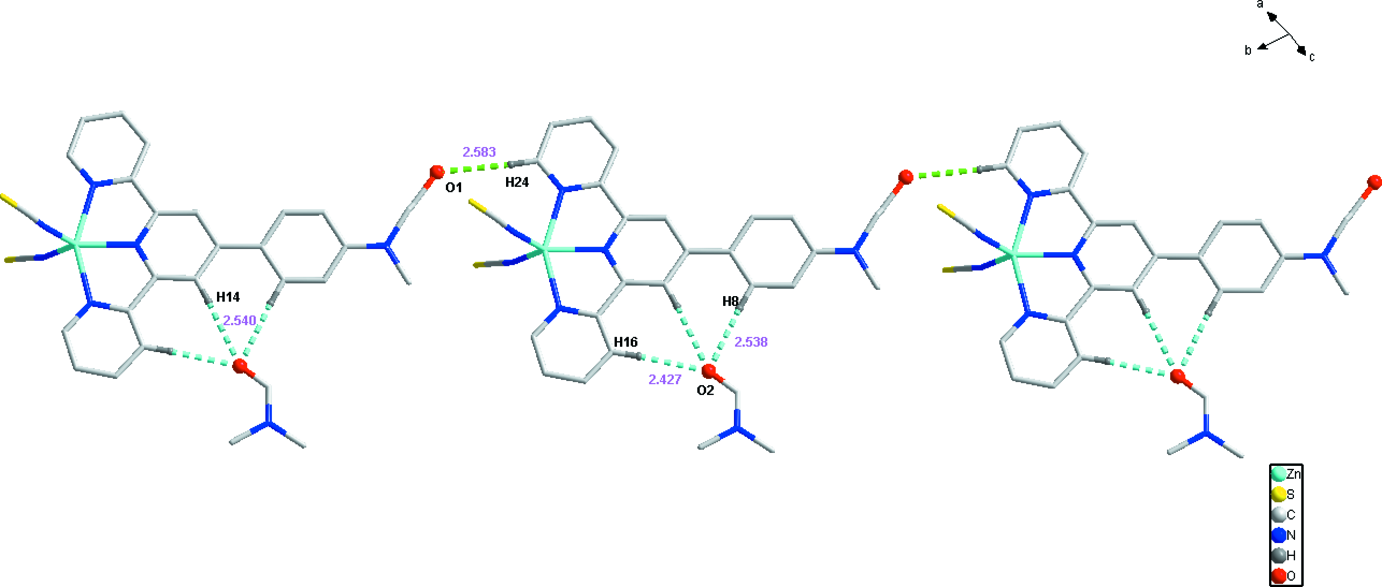

Supplement: Supplementary file 4 [file e-70-0m347-fig2.tif]

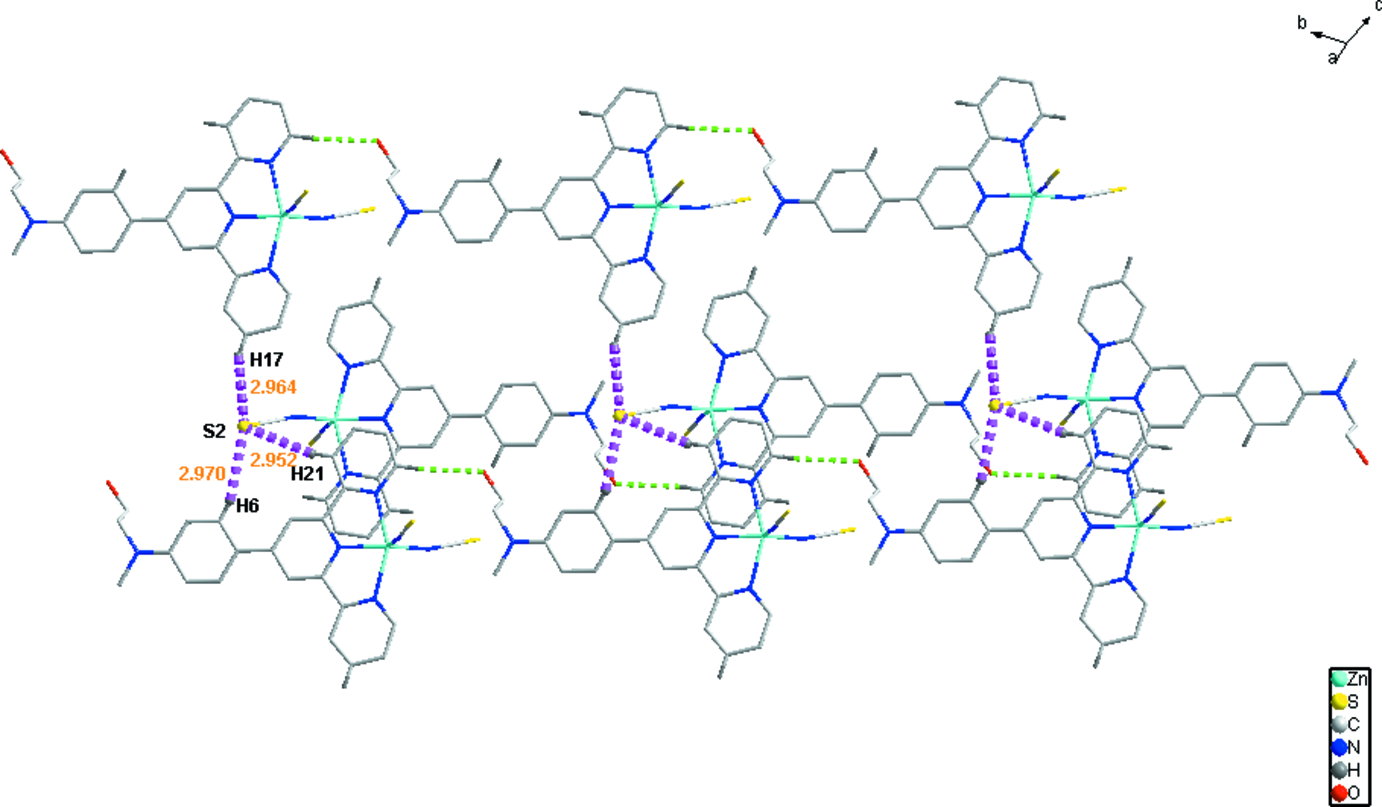

Supplement: Supplementary file 5 [file e-70-0m347-fig3.tif]
